# Maternal caffeine intake during pregnancy and child neurodevelopment up to eight years of age—Results from the Norwegian Mother, Father and Child Cohort Study

**DOI:** 10.1007/s00394-020-02280-7

**Published:** 2020-05-26

**Authors:** Sofia Berglundh, Margarete Vollrath, Anne Lise Brantsæter, Ragnhild Brandlistuen, Pol Solé-Navais, Bo Jacobsson, Verena Sengpiel

**Affiliations:** 1 Department of Obstetrics and Gynaecology, Sahlgrenska University Hospital, Gothenburg, Sweden; 2 Domain of Mental and Physical Health, Norwegian Institute of Public Health, Oslo, Norway; 3 Psychological Institute, University of Oslo, Oslo, Norway; 4 Section of Environmental Exposure and Epidemiology, Norwegian Institute of Public Health, Oslo, Norway; 5 Department of Child Health and Development, Norwegian Institute of Public Health, Oslo, Norway; 6 Department of Obstetrics and Gynaecology, Sahlgrenska Academy, University of Gothenburg, Gothenburg, Sweden

**Keywords:** MoBa, The Norwegian Mother, Father and Child Cohort Study, Maternal caffeine intake, Child neurodevelopment

## Abstract

**Purpose:**

Current knowledge of the effect of prenatal caffeine exposure on the child’s neurodevelopment is contradictory. The current study aimed to study whether caffeine intake during pregnancy was associated with impaired child neurodevelopment up to 8 years of age.

**Method:**

A total of 64,189 full term pregnancies from the Norwegian Mother, Father and Child Cohort Study were included. A validated food-frequency questionnaire administered at gestational week 22 was used to obtain information on maternal caffeine intake from different sources. To assess child neurodevelopment (behaviour, temperament, motor development, language difficulties) validated scales were used to identify difficulties within each domain at 6, 18, 36 months as well as 5 and 8 years of age. Adjusted logistic regression models and mixed linear models were used to evaluate neurodevelopmental problems associated with maternal caffeine intake.

**Results:**

Prenatal caffeine exposure was not associated with a persistently increased risk for behaviour, temperament, motor or language problems in children born at full-term. Results were consistent throughout all follow-ups and for different sources of caffeine intake. There was a minor trend towards an association between consumption of caffeinated soft drinks and high activity level, but this association was not driven by caffeine.

**Conclusion:**

Low to moderate caffeine consumption during pregnancy was not associated with any persistent adverse effects concerning the child’s neurodevelopment up to 8 years of age. However, a few previous studies indicate an association between high caffeine consumption and negative neurodevelopment outcomes.

**Electronic supplementary material:**

The online version of this article (10.1007/s00394-020-02280-7) contains supplementary material, which is available to authorized users.

## Background

Caffeine intake from different sources is common during pregnancy with coffee being the most frequent caffeine source in Scandinavian countries [1]. The Swedish National Food Agency recommends an intake of less than 300 mg caffeine per day during pregnancy [2], whereas the American College of Obstetricians and Gynaecologists advises a maximum intake of 200 mg per day [3]. Caffeine clearance is decreased during pregnancy [1] and metabolites pass the placenta [4] with possible accumulation of caffeine in the foetal brain [5]. An umbrella review from 2017 concluded that high coffee intake during pregnancy is associated with preterm birth, pregnancy loss and low birth weight [6]. Other studies have shown that maternal caffeine intake is associated with foetal growth restriction and increased risk of giving birth to a small for gestational age (SGA) baby [7, 8]. Children born SGA have an increased risk of cardiovascular disease later in life [9] and SGA has been related to impaired neurodevelopment during childhood [10, 11]. However, it is still unclear whether caffeine consumption during pregnancy affects the child’s neurodevelopment.

Caffeine metabolites are thought to increase vasoconstriction in the placenta [12], with possible negative effects on the foetus. Prenatal caffeine intake has been shown to have long lasting behavioural effects in rat offspring [13, 14]. The reported effect associated with prenatal caffeine intake in rodents is likely mediated by a reduction of acetylcholinesterase activity in the brain [15] and by effects on adenosine A receptors [16], and in the case of mice, by effects on GABA-neurons in hippocampus [17]. Although there is growing evidence that caffeine influences neurotransmitters in rodents, these findings cannot be directly transferred to humans.

Previous epidemiological studies suggest that maternal caffeine intake may affect child neurodevelopment, i.e. behaviour, motor development and language skills. However, previous findings are conflicting [18–24]. One study found an association between maternal caffeine intake and a higher risk of low IQ in 5.5-year old children [18]. Klebanoff et al. reported a J-shaped association between the caffeine metabolite paraxanthine and IQ at 7 years of age, but not at 4 years of age and they did not find a consistent association pattern for maternal caffeine intake and behavioural problems at any age [19]. A correlation between maternal coffee intake and social problems in children aged between four and nine has been reported, but there were only 19 participants consuming coffee in the study [24]. Other results indicate that prenatal caffeine intake does not increase the risk of behavioural problems in 5 to 6-year old children [22]. In line with this, Barr et al. did not find any association between caffeine and impaired neurobehavioral outcome in a 7-year prospective cohort study [21].

An association between maternal caffeine intake and inattention/over-activity in 18-months olds has been reported, but only for caffeine from soft drinks [20]. Linnet et al. showed that a very high intake of coffee (more than 10 cups/day) resulted in a three-fold increased risk for Attention Deficit Hyperactivity Disorder (ADHD), but the association lost significance after adjustment for confounders [23]. Another recent study also concluded that high coffee consumption (≥ eight cups per day) during pregnancy was associated with an increased risk of child hyperactivity-inattention disorder, however, the finding was not consistent for tea intake and the authors were not able to explore additional sources of caffeine exposure such as caffeinated soft drinks. Further, genetic aspects have been discussed as a possible confounder for the association found between caffeine consumption and hyperactivity [25].

Few studies have investigated motor and language development in association with prenatal caffeine exposure. Many of the previous studies have also had study design limitations, including few participants, only one caffeine source, follow-up at only one time point and limited data on confounders such as smoking and alcohol intake. Since current studies are contradictory and with important limitations, further studies are needed. The Norwegian Mother, Father and Child Cohort Study, with more than 108,000 included pregnancies, detailed information on caffeine intake from different sources, questionnaires on child follow-up based on validated instruments as well as comprehensive information on lifestyle and general health, is a unique source for investigating the association between prenatal caffeine exposure and child neurodevelopment. Currently, there is no study of comparable size with equally detailed information on exposure and covariates as well as long-term follow-up that can provide similarly robust data on the subject as this study.

## Aim

This study was aimed at investigating whether caffeine intake during pregnancy is related to neurodevelopment during childhood. The following aspects of development were studied: fine and gross motor development, language development, behaviour and temperament. We hypothesized that prenatal caffeine exposure negatively affects child neurodevelopment.

## Methods

### Study population

The study was based on the Norwegian Mother, Father and Child Cohort Study (MoBa) and the Medical Birth Registry of Norway (MBRN) [26, 27]. MoBa is a prospective population-based pregnancy cohort study conducted by the Norwegian Institute of Public Health. Participants were recruited from all over Norway from 1999 to 2008. The women consented to participation in 41% of the pregnancies. The cohort now includes 114,500 children, 95,200 mothers and 75,200 fathers. The current study is based on version 8 of the quality-assured data files released for research in 2015 [28]. All questionnaires are available at the website of the Norwegian Institute of Public Health [26].

### Inclusion and exclusion criteria

**Fig. 1:**
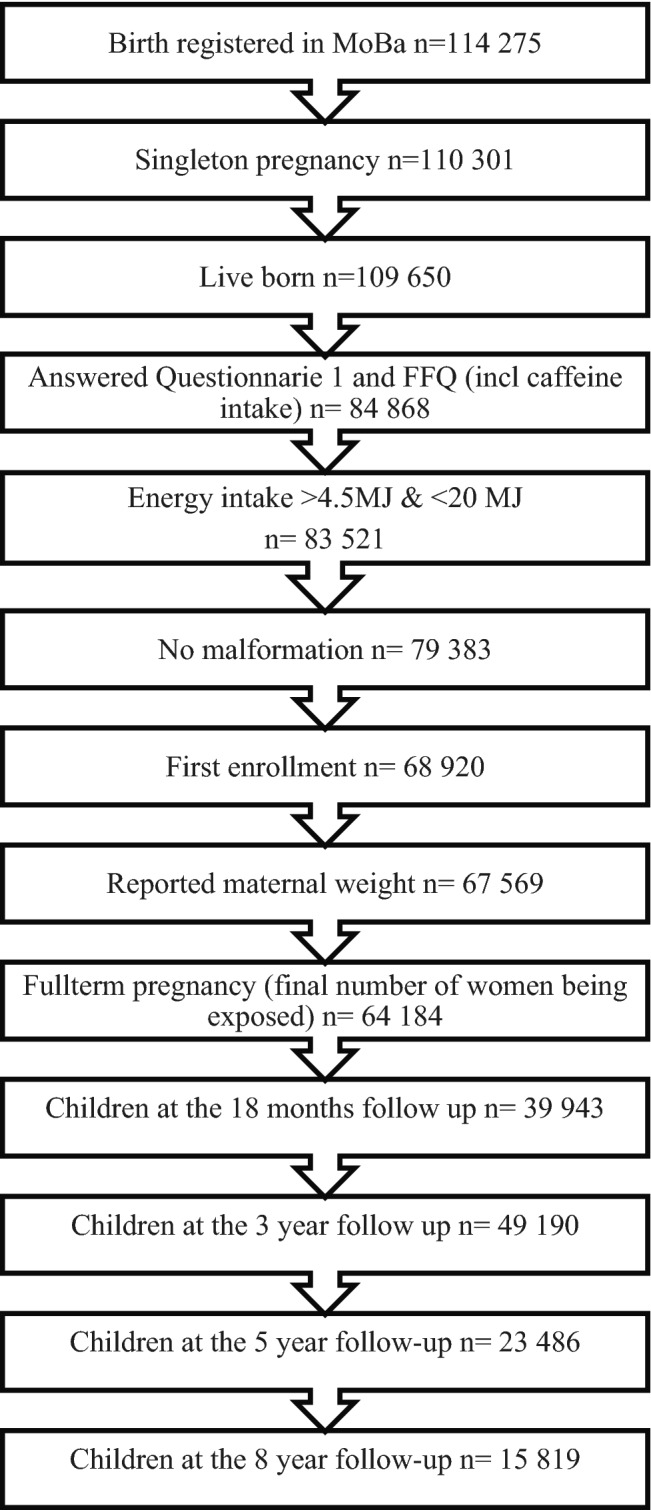
Flow chart inclusion and exclusion criteria and number of children at each follow-up. Women were recruited to MoBa between 1999 and 2008. The MoBa FFQ for assessment of caffeine intake was taken into use from 2002 and onwards, explaining the large proportion of missing information on caffeine intake (*n* = 24 782 (23%)) for live born singleton deliveries

Of 114,275 births registered in MoBa, all singleton, full-term pregnancies with live births between week 37 + 0 and 42 + 6 were included in the study. Further inclusion criterion was registration of caffeine intake. Children born with serious malformations were excluded. If a woman had participated in the study with more than one pregnancy, only the first pregnancy enrolled in MoBa was included in the analyses to avoid repeated measurements of the same mother. Caffeine intake was weight-adjusted; only participants with recorded weight data were thus included. Finally, as a quality measure, only women who reported an energy intake > 4.5 megajoules (MJ) and < 20 MJ were included. After exclusions 64,189 pregnancies remained. A flow-chart of the exclusion process is presented in Fig. 1, including the number of children at each follow-up time point.

### Exposure

Self-registered caffeine intake during the first half of pregnancy was used as the exposure variable. At gestational week 22, the women completed a Food Frequency Questionnaire (FFQ), in use from 2002 (explaining the gap between live births and reported caffeine intake, Fig. 1) [29]. The MoBa FFQ was developed specifically for assessing the intake of food, beverages and dietary supplements during pregnancy [30, 31]. The FFQ was validated in a subgroup of MoBa (*n* = 119) using a 4-day food diary as the reference, with high concordance for coffee (*r* = 0.80 CI 0.72–0.86). The detailed reporting of food and beverage intakes during the first half of the pregnancy enabled calculation of caffeine from different sources (coffee, tea, caffeinated beverages and chocolate), which was then summarized into total caffeine intake (mg/day) [7]. Both total caffeine intake and caffeine from different sources were analysed.

Caffeine intakes before pregnancy and at gestational weeks 17 (MoBa questionnaire 1) and 30 (MoBa questionnaire 3) were reported as cups/glasses of coffee/tea and caffeinated soft drinks per day. Caffeine intake was adjusted according to each participants pre-pregnancy body weight and recalculated as every woman weighed 65 kg, which corresponds to the median pre-pregnancy weight in the study population ((mg/day × 65)/(pre-pregnancy weight)). Weight adjustment is common when examining health effects of toxicants, including caffeine [32].

### Outcome

The main outcome in this study was the child’s neurodevelopment at different ages (6 and 18 months as well as 3, 5 and 8 years) assessed with questionnaire data, provided by the mothers, focusing on the child’s behaviour and temperament and motor and language development. Key items for each questionnaire had been selected from standardized, validated scales widely used for assessing child neurodevelopment and behaviour and assembled into shortened scale versions for the MoBa cohort [33–46]. (Detailed information is available in the Online Appendix).

Items from the following scales were used:

- Behaviour
  - The infant characteristics questionnaire, fussy/difficult subscale (ICQ) [33] (6 months).
  - Child behaviour checklist (CBCL) [34] (18 months, 3 and 5 years).
  - Infant–Toddler social and emotional assessment (ITSEA) [35] (3 years].
  - Emotionality, activity and shyness temperament questionnaire (EAS) [36] (18 months, 3, 5 years).
  - The short moods and feeling questionnaire [37, 38] (8 years).
  - Screen for child anxiety-related disorders (SCARED) [39] (8 years).
  - Parent/teacher rating scales for disruptive behaviours (RS-DBD) [41] (8 years).
- Development
  - The ages and stages questionnaire (ASQ) [42, 43] (18 months, 3 and 5 years).
  - The child development inventory (CDI) [44] (5 years).
  - The children’s communication checklist-2 (CCC-2) [45, 46] (8 years).

Questionnaire data were summarized and a mean value was calculated for each scale. Cut-offs were set at > 1.5 standard deviation (SD) above mean, (or below 1.5 SD for sociability which is considered a positive trait). Cut-off for activity level at 18 months was set at > 1 SD, since > 1.5 SD would yield 0 cases. The number of children with a value above mean score + 1.5 SD for the different neurodevelopment outcomes are presented in Table 1.

**Table 1:** Number and percentage of children with a value above mean score + 1.5 SD for the different neurodevelopment outcomes at follow-up

	18 months		3 years		5 years		8 years	
	*N*	%	*N*	%	*N*	%	*N*	%
Low sociability	4248	8.7	2362	5.9	1529	6.5		
Negative emotionality	3505	7.2	3486	8.8	2311	9.9		
Shyness	4887	7.1	3420	8.6	1570	6.7		
High activity*	5470	11.2	1730	4.3	2540	10.9		
Internalizing behaviour	3540	7.2	3255	8.2	2103	9.1		
Externalizing behaviour	4191	8.5	2891	7.3	860	3.7		
Fine motor	5704	11.7	4100	10.4	1742	7.5		
Gross motor	3726	7.6	1252	3.1	1314	5.6		
Language development	4706	9.6	2015	5.1	1613	7.0	1323	7.8
Motor development							201	0.3
Conduct disorder							946	6.0
ADHD-related symptoms							1136	7.2
Oppositional defiant							1342	8.5
Depression symptoms							1776	11.3
Scared/anxiety							1472	9.3

*At 18 months > mean + 1 SD

### Confounders

The following confounders were chosen prior to the analyses, due to their relevance for exposure and outcome. Information about maternal age (years) at delivery and the baby’s sex was obtained from the Medical Birth registry. From the first MoBa questionnaire we obtained information about household income (both parents had income < 300,000 Norwegian crowns (NOK)/year, one of the parents had income ≥ 300,000 NOK, both parents had income ≥ 300,000 NOK or missing), maternal education level (number of years school attendance: ≤ 12, 13–16, ≥ 17 years or missing), marital status (cohabiting or not), and smoking (daily, occasionally, never). Intake of alcohol (units per week: none, < 0.5 unit/w, ≥ 0.5 unit/w), nausea during pregnancy (yes/no), energy intake (kJ) and dietary fibre (included as a proxy of a healthy diet) were obtained from the MoBa FFQ. Furthermore, from the first MoBa questionnaire we also obtained maternal depression symptoms based on a short version of the Hopkins Symptom Checklist, and information about pre-pregnancy maternal weight and height for calculating BMI. BMI was categorised according to the WHO classification as underweight (< 18.5 kg/m^2^), normal weight (18.5–24.9 kg/m^2^), overweight (25.0–29.9 kg/m^2^) and obese (≥ 30.0 kg/m^2^).

### Ethics

The establishment and data collection in MoBa have obtained a licence from the Norwegian Data Inspectorate and approval from The Regional Committee for Medical Research Ethics (S-95113, and (S-97045). The Regional Committee for Medical Research Ethics has approved the current study (2010/2683/REK sør-øst A). Informed consent has been collected from all participants.

### Statistics

Caffeine intake stratified by maternal baseline characteristics (as described under confounders) was compared using the Kruskal–Wallis or Mann–Whitney *U* test as appropriate. Baseline characteristics were also compared between responders and non-responders at the 3 and 8-year follow-ups. Caffeine intake was analysed continuously as well as according to categories in order to test for possible threshold effects: 0–22 (corresponding to the lowest quartile), > 22–56 (corresponding to the second lowest quartile), > 56–200, > 200–300 and > 300 mg/day (the three latter categories based on existing recommendations).

Logistic regression models were used to calculate odds ratios (OR) for outcome (i.e. behavioural or temperamental problem, motor or language difficulties) and were adjusted for confounders. Linear mixed effects models were fitted for four of the outcomes (negative emotionality, high activity, shyness and low sociability) with repeated measurements available at 18 and 36 months and 5 years. Here, we employed continuous *z*-scores calculated at each measurement as the outcomes. The crude model included maternal caffeine intake and age at child measurement as fixed effects, and we allowed for separate intercepts for each subject by including subject identifier as random effect. The adjusted models included the above-mentioned confounders as fixed effect. Models were fitted by restricted maximum likelihood estimation using ‘lmer’ function from the ‘lme4’ R package (version 3.4.3). A similar analytical approach was not applicable to the other outcomes since the measurements varied between the different time points (e.g. disparities between the questionnaires).

Sensitivity analyses were performed for boys and girls separately in order to identify potential sex-specific effects as well as for different sources of caffeine (coffee, tea, caffeinated soft drinks, chocolate), all mutually adjusted. Furthermore, in order to investigate whether there is a time period during pregnancy when the developing foetal brain is more vulnerable to potential caffeine effects, pre-pregnancy caffeine exposure and exposures at gestational weeks 17 and 30 were explored in a sub-analysis using logistic regression models.

Analyses were performed in SPSS version 23.0 and 24.0 (IBM Corp. Armonk, NY, USA) and in R (version 3.4.3) for the mixed linear models.

## Results

### Maternal characteristics

**Fig. 2:**
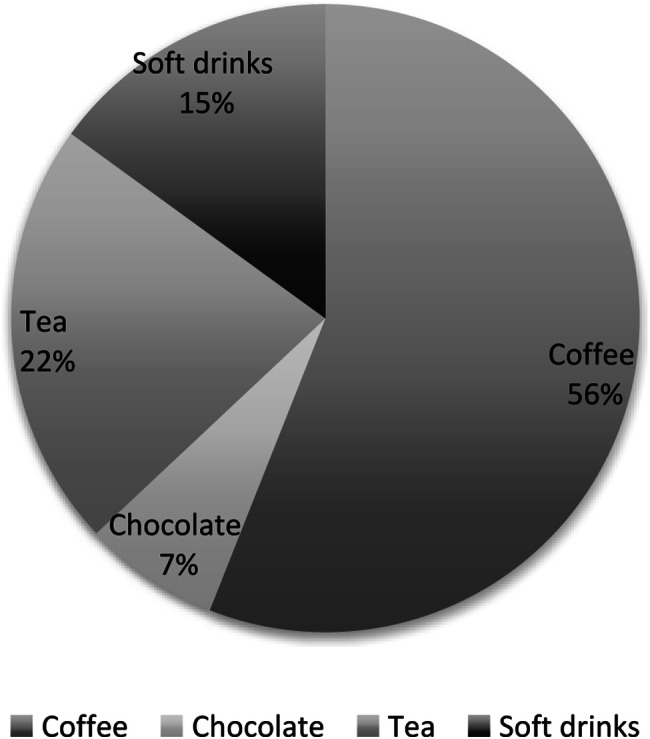
Percentage of total caffeine intake per caffeine source

Most women had a low consumption of caffeine with only 7.4% consuming > 200 mg/day and 3.5% consuming > 300 mg/day. Caffeine intake was skewed to the right and the median intake was 56 mg/day (approximately ½ cup per day) at mid-pregnancy. The total caffeine intake differed between the various time points, with the highest intake before pregnancy (median 172 mg/day), the lowest in mid-pregnancy (median 56 mg/day) and then increasing again in the last trimester (median 80 m/day) (Supplementary Table 1). Coffee was the most common caffeine source representing 56% of the total intake (Fig. 2). Caffeine intake was higher for women with a high education level, who were smokers, consumed alcohol and were older (Table 2). Nausea was associated with reduced caffeine intake (Table 2).

**Table 2:** Total caffeine and caffeine intake from coffee according to maternal characteristics, *n* = 64 189 from the Norwegian Mother and Child Cohort

Characteristic	Category	%	Count	Total caffeine intake (mg/day)	Caffeine intake from coffee (mg/day)
				Median (25th; 75th percentile)	Median (25th; 75th percentile)
Total cohort			64,189	56 (99)	7 (0; 68)
Maternal age at delivery (years)*	< 25	11.3	7263	47 (19; 102)	0 (0; 14)
	25–29	34.0	21,833	34 (15; 82)	4 (0; 45)
	30–34	42.6	27,357	67 (27; 133)	11 (0; 82)
	35 +	12.1	7736	89 (38; 169)	27 (0; 122)
Maternal education level (years)*	≤ 12	30.6	19,638	54 (21; 128)	4 (0; 64)
	13–16	41.8	26,833	53 (21; 114)	7 (0; 62)
	17 +	25.5	16,358	66 (27; 126)	14 (0; 79)
	Missing data	2.1	1360	60 (22; 123)	7 (0; 71)
Marital status	Married/cohabitant	96.2	61,750	57 (22; 120)	7 (0; 68)
	not Married/cohabitant	3.8	2439	59 (21; 141)	6 (0; 75)
Alcohol intake (units/week)*	No	89.0	57,107	53 (21; 114)	6 (0; 59)
	< 0.5	9.2	5929	94 (44; 169)	38 (4; 117)
	> 0.5	1.8	1153	125 (66; 216)	74 (13; 174)
Smoking habits*	Never	92.0	58,709	54 (21; 113)	6 (0; 60)
	Occasionally	2.7	1731	107 (41; 199)	43 (0; 162)
	Daily	5.3	3387	144 (56; 260)	71 (0; 197)
Nausea during pregnancy*	No	88.7	56,946	59 (23;125)	8 (0; 74)
	Yes	11.3	7243	43 (16; 96)	0 (0; 21)
BMI prior to pregnancy (kg/m^2^)*	< 18.5	3.0	1886	62 (24; 128)	9 (0; 86)
	18.5–24.9	66.2	42,309	73 (29; 151)	9 (0; 78)
	24.9–29.9	21.6	13,794	51 (20; 107)	5 (0; 57)
	30+	9.3	5914	38 (14; 84)	2 (0; 30)
Income of ≥ 300,000 NOK/year*	None in the household	27.6	17,705	49 (19; 113)	4 (0; 53)
	One in the household	41.1	26,368	56 (22; 121)	6 (0; 67)
	Both in the household	28.6	18,326	67 (27; 128)	13 (0; 82)
	Missing data	2.8	1790	55 (20; 125)	4 (0; 59)
Tertiles of fiber intake*	1	33.3	21,394	49 (19; 106)	4 (0; 49)
	2	33.3	21,388	58 (23; 120)	8 (0; 70)
	3	33.3	21,397	67 (26; 139)	10 (0; 83)
Sex of baby	Boy	50.7	32,572	57 (23; 122)	7 (0; 68)
	Girl	49.3	31,617	57 (22; 120)	7 (0; 68)

*BMI* body mass index, *NOK* Norwegian kroner, *IQR* interquartile range

*Statistically significant difference between the groups

The total number of respondents decreased over the follow-up period with a response rate of 77% (*n* = 49 190) at 18 months, 62% (*n* = 39 943) at 3 years, 37% (*n* = 23 486) at 5 years and 24% (*n* = 15 819) at the 8-year follow-up. At the 8-year follow-up, only around 60% of the study population were eligible (i.e. the child had reached 8 years of age) when collecting the data for the analyses in this study, explaining the low response rate. At the 3-year follow-up, the non-responders had a lower education level, lower income and a somewhat lower reported alcohol intake and were more frequently daily smokers. The median caffeine intake was slightly higher for non-responders (median 59 vs. 55 mg/day) (Table 3). At the last follow-up (8 years) there were more participants with a high education level (> 17 years) and high income among the non-responders. Furthermore, the non-responders were more frequently smokers, but the caffeine intake compared to the responders was rather similar (median 57 vs. 55 mg/day). The mean values for child outcome and percentages of children with a mean score > 1.5 SD at 18 months are presented in Table 4 for responders and non-responders at the 3-year follow-up, respectively.

**Table 3:** Median (IQR) caffeine intake at baseline for responders and non-responders at different follow-ups

	Median caffeine intake	
	Responders	Non responders
18 months follow -up	56 (94)	56 (98)
3 year follow-up	55 (96)	59 (104)
5 yr follow-up	63 (105)	60 (107)
8 yr follow-up	57 (98)	55 (99)

*IQR* inter quartile range

**Table 4:** Mean (SD) value for child outcomes at 18 months and percentages (%) of children above the cut-off value for responders and non-responders at the 3-year follow-up

	Responders at the 3-year follow-up		Non-responders at the 3-year follow-up	
18 months	Mean (SD)	% of children with value > mean + 1.5 SD	Mean (SD)	% of children with value > mean + 1.5 SD
Low sociability (EAS)	3.96 (0.56)	8.8%	3.96 (0.56)	8.4%
Negative emotionality (EAS)	2.73 (0.76)	7.3%	2.74 (0.76)	6.7%
Shyness (EAS)	2.04 (0.64)	10.1%	2.03 (0.64)	9.7%
High activity (EAS)	4.03 (0.65)	11.3%	4.04 (0.64)	11.0%
Internalizing behaviour (CBCL)	1.25 (0.24)	6.7%	1.26 (0.25)	8.2%
Externalizing behaviour (CBCL)	1.49 (0.30)	8.4%	1.49 (0.31)	8.9%
Fine motor (ASQ)	1.13 (0.27)	11.8%	1.13 (0.27)	11.4%
Gross motor (ASQ)	1.10 (0.28)	7.8%	1.10 (0.28)	7.1%
Language development (ASQ)	1.40 (0.50)	9.7%	1.39 (0.50)	9.5%

*SD* standard deviation

### Child’s behavioural and emotional outcomes

Mixed linear models, taking 3 time-points into consideration (18 months, 3 and 5 years of age), showed a small association between total caffeine intake and low sociability, negative emotionality and high activity (beta coefficient between 0.01 and 0.02 per 100 mg caffeine intake, 95% CI 0.002–0.030) (Table 5). The correlation seen for high activity level at 18 months to 5 years of age was weakly related to caffeine intake from coffee (OR between 1.039 and 1.076, 95% CI 1.004–1.133), but more strongly to caffeine from soft drinks (OR between 1.240 and 1.443, 95% CI 1.125–1.671) (Supplementary Table 2).

**Table 5:** Linear mixed effects model for child temperament outcomes at different ages between 18 months and 5 years of age according to total caffeine intake

	Negative emotionality		High Activity		Shyness		Low Sociability	
	Beta	95% CI	Beta	95% CI	Beta	95% CI	Beta	95% CI
Crude model	**0.013**	0.005, 0.021	**0.020**	0.012, 0.028	**−0.011**	−0.019, −0.003	**0.010**	0.002, 0.017
Full model	**0.010**	0.002, 0.019	**0.022**	0.013, 0.030	−0.008	−0.016, 0.001	**0**.**020**	0.012, 0.028

Beta and 95% CI for negative emotionality, high activity, shyness and low sociability using linear mixed effects models according to total maternal caffeine (per 100 mg increased intake). Number of measurements and sample size varied with outcome, ranging between 112,003 and 111,329, and 53,005 and 52,681, respectively

Crude model included maternal caffeine intake and child age at measurement as fixed effects. Full model was adjusted for: maternal age, smoking, alcohol intake, marital status, baby’s gender, household income, maternal education, dietary fiber, total energy intake, nausea, maternal mental health, all included as fixed effects. *CI* confidence interval. Statistically significant results are in bold. The outcomes are based on the EAS scales

There was no consistent association between total maternal caffeine intake and an increased risk of child behavioural problems between 18 months and 5 years of age (Table 6).

**Table 6:** Adjusted odds ratios for child behavior outcomes at different ages between 18 months and 5 years of age according to total maternal caffeine intake and category of caffeine intake

Caffeine intake (mg/day)	Internalizing Behaviour		Externalizing Behaviour	
**18 months**	OR	95% CI	OR	95% CI
Total caffeine intake (100 mg/day)	1.001	0.962–1.041	0.995	0.959–1.032
Caffeine intake 0–22 (*n* = 12317)	Ref		Ref	
Caffeine intake > 22–56 (*n* = 12605)	0.956	0.867–1.054	1.005	0.917–1.101
Caffeine intake > 56–200 (*n* = 19201)	**0.909**	0.829–0.996	1.009	0.926–1.100
Caffeine intake > 200–300 (*n* = 3563)	0.976	0.842–1.131	1.097	0.968–1.257
Caffeine intake > 300 (*n* = 1504)	1.017	0.828–1.250	0.921	0.755–1.123
**3 years**	OR	95% CI	OR	95% CI
Total caffeine intake (100 mg/day)	0.976	0.934–1.019	1.030	0.987–1.075
Caffeine intake 0–22 (*n* = 10082)	Ref		Ref	
Caffeine intake > 22–56 (*n* = 10275)	1.012	0.913–1.120	0.991	0.889–1.105
Caffeine intake > 56–200 (*n* = 15550)	1.003	0.911–1.105	0.975	0.880–1.081
Caffeine intake > 200–300 (*n* = 2833)	0.932	0.792–1.097	1.059	0.898–1.248
Caffeine intake > 300 (*n* = 1203)	0.977	0.778–1.228	1.166	0.934–1.455
**5 years**	OR	95% CI	OR	95% CI
Total caffeine intake (100 mg/day)	0.962	0.910–1.018	1.007	0.956–1.061
Caffeine intake 0–22 (*n* = 5716)	Ref		Ref	
Caffeine intake > 22–56 (*n* = 6135)	**0.854**	0.751–0.971	**0.869**	0.766–0.985
Caffeine intake > 56–200 (*n* = 9280)	0.898	0.797–1.012	**0.870**	0.774–0.979
Caffeine intake > 200–300 (*n* = 1706)	0.837	0.685–1.022	0.917	0.758–1.110
Caffeine intake > 300 (*n* = 649)	0.833	0.616–1.127	1.033	0.789–1.352

Odds ratio for behavior outcomes during childhood at different ages according to total caffeine (per 100 mg increased intake) and different categories of caffeine intake. Caffeine category 1: 0–22 (reference), 2: > 22–56, 3: > 56–200, 4: > 200–300, 5: > 300 mg/day. Adjusted for: maternal age, smoking, alcohol intake, marital status, baby’s gender, household income, maternal education, dietary fiber, total energy intake, nausea, maternal mental health. *OR* odds ratio, *CI* confidence interval. OR (CI) from logistic regression. Statistically significant results are in bold. The outcomes are based on the CBCL scale

Total maternal caffeine intake and caffeine intake from different sources were not associated with a higher risk of child conduct problems, ADHD symptoms, oppositional defiant behaviour, depression symptoms or anxiety at 8 years of age (Table 7).

**Table 7:** Adjusted Odds Ratios for child temperament and behavior outcomes at 8 years of age according to total maternal caffeine intake and category of caffeine intake

8 years	Conduct disorder		ADHD-symptoms		Oppositional defiant		Depression symptoms		Scared/anxiety	
	OR	95% CI	OR	95% CI	OR	95% CI	OR	95% CI	OR	95% CI
Total caffeine intake (100 mg/day)	1.027	0.956–1.103	1.025	0.960–1.096	1.015	0.953–1.081	1.042	0.986–1.101	0.975	0.916–1.039
Caffeine intake 0–22 (*n* = 4112)	Ref		Ref		Ref		Ref		Ref	
Caffeine intake > 22–56 (*n* = 3959)	0.917	0.760–1.107	0.967	0.814–1.150	0.958	0.820–1.120	0.917	0.794–1.058	1.016	0.873–1.182
Caffeine intake > 56–200 (*n* = 6025)	0.862	0.720–1.032	0.873	0.739–1.031	**0.837**	0.719–0.973	0.932	0.814–1.066	0.955	0.827–1.102
Caffeine intake > 200–300 (*n* = 1159)	1.127	0.859–1.480	1.045	0.806–1.357	0.958	0.751–1.222	1.065	0.860–1.317	0.987	0.781–1.247
Caffeine intake > 300 (*n* = 564)	0.948	0.655–1.372	1.125	0.809–1.564	1.194	0.881–1.618	1.281	0.978–1.677	0.956	0.694–1.316

Odds ratio for behavior and temperament outcomes during childhood at 8 years of age according to total caffeine (per 100 mg increased intake) and different categories of caffeine intake. Caffeine category 1: 0–22 (reference), 2: > 22–56, 3: > 56–200, 4: > 200–300, 5: > 300 mg/day. Adjusted for: maternal age, smoking, alcohol intake, marital status, baby’s gender, household income, maternal education, dietary fiber, total energy intake, nausea, maternal mental health. *OR* odds ratio, *CI* confidence interval. OR (CI) from logistic regression. Statistically significant results are in bold. The outcomes are based on the SMFQ, SCARED and RS-DBD scales

Caffeine intake from chocolate was associated with child fussiness at 6 months of age (OR = 2.049, 95% CI 1.464–3.963) and negative emotionality at 18 months (OR = 2.745, 95% CI 1.565–4.816) (Supplementary Tables 2 and 4).

### Child’s motor and language development

There was an association between caffeine intake and gross motor impairment at age 18 months (OR = 1.055 95% CI 1.016–1.095, Table 8). No such association was found at 3 and 5 years of age for total caffeine intake or caffeine from different sources (Supplementary Table 3). At the 8-year follow-up, the number of participants reporting current or previous motor development problems was low (*n* = 201) and no risk increase was found for any of the caffeine sources. There was no persistent association between total caffeine intake and fine motor development between the follow-ups.

**Table 8:** Adjusted odds ratios for child language and motor difficulties at different ages between 18 months and 8 years of age according to total maternal caffeine intake and category of caffeine intake

Caffeine intake (mg/day)	Gross motor		Fine motor		Language	
18 months	OR	95% CI	OR	95% CI	OR	95% CI
Total caffeine intake (100 mg/day)	**1.055**	1.016–1.095	0.990	0.958–1.022	**1.043**	1.008–1.079
Caffeine intake 0–22 (*n* = 12317)	Ref		Ref		Ref	
Caffeine intake > 22–56 (*n* = 12605)	1.058	0.958–1.168	0.945	0.873–1.022	0.978	0.894–1.070
Caffeine intake > 56–200 (*n* = 19201)	**1.180**	1.078–1.293	1.006	0.935–1.083	**1.102**	1.015–1.197
Caffeine intake > 200–300 (*n* = 3563)	**1.174**	1.016–1.357	**0.880**	0.778–0.996	1.085	0.952–1.236
Caffeine intake > 300 (*n* = 1504)	**1.299**	1.063–1.588	0.945	0.796–1.123	1.107	0.921–1.330
3 years	OR	95% CI	OR	95% CI	OR	95% CI
Total caffeine intake (100 mg/day)	0.941	0.875–1.012	0.963	0.924–1.003	1.004	0.970–1.073
Caffeine intake 0–22 (*n* = 10082)	Ref		Ref		Ref	
Caffeine intake > 22–56 (*n* = 10275)	1.062	0.907–1.243	0.925	0.842–1.016	0.947	0.831–1.079
Caffeine intake > 56–200 (*n* = 15550)	0.962	0.827–1.119	0.953	0.874–1.040	1.033	0.916–1.166
Caffeine intake > 200–300 (*n* = 2833)	1.005	0.783–1.288	0.972	0.841–1.124	0.997	0.818–1.214
Caffeine intake > 300 (*n* = 1203)	0.891	0.604–1.314	0.817	0.653–1.023	1.158	0.895–1.499
5 years	OR	95% CI	OR	95% CI	OR	95% CI
Total caffeine intake (100 mg/day)	0.978	0.911–1.049	0.996	0.939–1.057	0.960	0.902–1.021
Caffeine intake 0–22 (*n* = 5716)	Ref		Ref		Ref	
Caffeine intake > 22–56 (*n* = 6135)	0.953	0.813–1.118	0.897	0.776–1.037	**0.848**	0.733–0.981
Caffeine intake > 56–200 (*n* = 9280)	0.953	0.821–1.107	1.005	0.879–1.149	0.901	0.787–1.030
Caffeine intake > 200–300 (*n* = 1706)	0.952	0.744–1.219	1.087	0.880–1.343	0.821	0.655–1.029
Caffeine intake > 300 (*n* = 649)	0.988	0.679–1.439	0.763	0.543–1.073	0.888	0.644–1.224
	Motor				Language	
8 years	OR	95% CI			OR	95% CI
Total caffeine intake (100 mg/day)	0.968	0.832–1.127			0.986	0.927–1.049
Caffeine intake 0–22 (*n* = 4112)	Ref				Ref	
Caffeine intake > 22–56 (*n* = 3959)	1.174	0.777–1.774			1.017	0.868–1.192
Caffeine intake > 56–200 (*n* = 6025)	1.229	0.837–1.804			0.981	0.845–1.138
Caffeine intake > 200–300 (*n* = 1159)	1.048	0.567–1.937			**0.754**	0.581–0.977
Caffeine intake > 300 (*n* = 564)	0.726	0.295–1.786			0.911	0.661–1.255

Odds ratio for language and motor difficulties during childhood at different ages according to total caffeine (per 100 mg increased intake) and different categories of caffeine intake. Caffeine categories: 0–22 (ref), > 22–56, > 56–200, > 200–300, > 300 mg/day. Adjusted for: maternal age, smoking, alcohol intake, marital status, baby’s gender, household income, maternal education, dietary fiber, total energy intake, nausea, maternal mental health. *OR* odds ratio, *CI* confidence interval. OR (CI) from logistic regression. Statistically significant results are in bold. The outcomes are based on the ASQ, CDI and CCC-2 scales

Caffeine intake was weakly associated with language difficulties at 18 months (OR = 1.043, 95% CI 1.008–1.079), but the association did not remain at 3, 5 or 8 years of age (Table 8). Analyses of caffeine from different sources, mutually adjusted, did not show an association with language difficulties except for caffeine intake from tea at the 18-month follow-up (OR = 1.241, 95% CI 1.126–1.367, Supplementary Table 3).

Investigation of caffeine intake according to categories showed a trend towards an increased risk of gross motor impairment for total caffeine intake > 56–200 mg/day (OR = 1.180, 95% CI 1.078–1.293), > 200–300 mg/day (OR = 1.174, 95% CI 1.016–1.357) and > 300 mg/day (OR = 1.299, 95% CI 1.063–1.588) at the 18-month follow-up.

### Sensitivity analyses

#### Caffeine intake at different time-points

Caffeine intake at gestational week 30 was associated with externalizing behaviour at 18 months of age (OR = 1.040, 95% CI 1.020–1.060) and depression symptoms at 8 years of age (OR = 1.044, 95% CI 1.006–1.084). Caffeine intake at gestational week 17 was associated with gross and fine motor difficulties at 18 months of age (OR = 1.024 95% CI 1.003–1.057), respectively (OR = 1.032, 95% CI 1.007–1.057). Pre-pregnancy caffeine intake was associated with language development at the 8-year follow-up. (Supplementary Tables 5 and 6).

#### Soft drinks

To explore whether the association between caffeinated soft drinks and activity level was related to the caffeine component, analyses were performed for non-caffeinated and caffeinated soft drinks separately. The analyses did not reveal a difference between caffeinated and non-caffeinated soft drinks. In an unadjusted model, the OR for high activity level was 1.011–1.018 (95% CI 1.008–1.023) for caffeinated soft drinks and 1.009–1.017 (95% CI 1.003–1.027) for non-caffeinated soft drinks (Supplementary Table 7). Smokers and participants with low education level consumed soft drinks more frequently.

#### Sex-specific analyses

Sex-specific analyses revealed that the observed tendency towards high activity was more consistent throughout the years for girls (all time-points) compared to boys (only at 5 years). No other significant differences were found (data not shown).

## Discussion

In this study, a potential association between caffeine intake during pregnancy and the child’s neurodevelopment at different ages was explored. The results indicate that low to moderate caffeine exposure during pregnancy is not associated with any consistent adverse effect on the child’s neurodevelopment between 6 months and 8 years of age for children born at term.

The use of mixed linear model was possible for the emotionality outcomes and showed an association between total caffeine intake and high activity, low sociability and negative emotionality. However, the effect sizes were very small with corresponding beta coefficients between 0.01 and 0.02 per 100 mg increase in caffeine intake. The analyses allowed the use of all data available regarding the child’s emotionality, by including all time points and yielded a high number of cases. The very small effect of caffeine can hardly be considered to be of clinical relevance but merely a statistical association due to a high number of included observations [47]. Unfortunately, similar analyses were not possible for the other outcomes due to the discrepancies between the questionnaires at different time points.

The tendency towards an association between prenatal caffeine exposure and high activity seemed to be driven mainly by soft drink consumption. Investigations separating non-caffeinated and caffeinated soft drinks yielded the same results, indicating that it is not the caffeine component in soft drinks that causes the association. Our findings are in line with previous data. A previous study from the MoBa concluded that a high prenatal intake of soft drinks, but not total caffeine exposure, increased the risk of cerebral palsy (HR 1.9, 95% CI 1.2–3.1) [48]. Another study from the MoBa cohort showed an association between caffeine intake during pregnancy and inattention/over-activity symptoms, but only related to caffeinated soft drinks **[**20]. This further supports our hypothesis that the association found for soft drinks in the current study was not caused by caffeine per se.

Caffeine from chocolate was associated with fussiness and negative emotionality at 6 and 18 months, respectively. These findings are probably due to residual non-nutritional confounding or to some other substance in chocolate, since they did not follow a clear pattern at the different follow-ups, nor were there similar associations concerning other caffeine sources. The effect size seems rather large, but one should bear in mind that due to the relatively low content of caffeine in chocolate, consumption of 100 mg of caffeine from chocolate requires a considerable consumption of chocolate.

Total caffeine intake was weakly related to language difficulties at 18 months, but the association disappeared at the 3-year follow-up. When investigating different sources of caffeine, caffeine from tea was associated with language difficulties at 18 months but no comparable association was found for caffeine from coffee. To our knowledge, there are no previous studies examining the association of prenatal caffeine exposure with language development.

At 18 months, there was an association between increasing caffeine exposure and gross motor development, however, the effect size was small. Although it is possible that the association found at 18 months could be a caffeine effect, there were no persistent consequences for the child as there was no association at three, 5 or 8 years of age. A previous study has reported a lack of association between caffeine and motor development at 4 years of age, supporting the robustness of our findings [21].

We performed sensitivity analyses to explore the effect of caffeine intake at different time-points during pregnancy. Caffeine intake at gestational week 30 was associated with externalizing behaviour at 18 months and depression symptoms at 8 years. These findings might be related to a specific source of caffeine intake or might be explained by residual confounding. With regards to the high number of statistical analyses performed in this study, some of the small associations seen are probably due to chance. However, we cannot fully discard the possibility that there is a true association. There were also associations between caffeine exposure at week 17 and motor development at 18 months, but not at the later time points, similar to total caffeine intake in the main analysis. Overall, our findings do not suggest that there is a specific period during pregnancy in which moderate caffeine exposure is more hazardous with regard to neurodevelopment. However, the MoBa participants’ caffeine consumption was to a large degree below current recommended maximum, which limits the power to detect a potential effect related to high caffeine consumption (> 300 mg/day).

Our data indicates that soft drinks consumed during pregnancy may have a negative effect on the child. We cannot rule out a potential effect from other components in soft drinks or that the association is due to confounding by other lifestyle habits. Since a link between intake of soft drinks and an increased risk of pre-term birth has previously been reported [49], it might be of interest to further explore the potential consequences for the child’s health related to other components in soft drinks.

In our previous study, caffeine was shown to be associated with lower birth weight and increased risk of SGA [7]. However, our results do not support the hypothesis that caffeine has a persistent negative effect on the child’s neurodevelopment. Similar results have been found for smoking where children being born SGA related to smoking may have better outcomes compared to those born SGA related to other causes [50]. Nevertheless, regardless of the negative findings in this study regarding neurocognitive outcome, others have shown harmful association of high coffee intake with other important outcomes, for example preterm births [6], and the current safety guidelines during pregnancy of an intake of 200 mg/day should be kept in mind [32].

### Strengths and limitations

To our knowledge, this is the most comprehensive study on the association between prenatal caffeine exposure and neurodevelopment during childhood. Observational studies are the best option for studying associations between caffeine exposure and these outcomes, in spite of inherent limitations such as selection bias, confounding and loss to follow-up.

In this study, the number of participants was high but the overall participation rate in the MoBa is low (41%) [28]. The number of subjects lost to follow-up increased over the study period. Nonetheless, the large number of participants still yielded a fair number of cases. At the 3-year follow-up, the non-responders had lower education and were more frequent smokers. However, at the last follow-up (8 years) the non-responders generally had a higher education level and income, but similar outcomes were found as for the earlier time points. Median caffeine intake comparison between non-responders and responders displayed a difference of 2–4 mg, which was not considered of relevance.

As seen in Table 3, the percentages of children above the cut-off value at 18 months are similar for responders and non-responders at the 3-year follow-up for all outcomes except for internalizing behaviour (6.7% vs. 8.2%). The overall small disparities between the groups suggest that attrition bias is not a major concern.

Caffeine intake was self-reported, which might have resulted in under- or overestimation of caffeine intake. However, the data collection was prospective, minimizing the risk of recall bias. Coffee intake is easier to recall than most other food intakes and the validation study of the FFQ reported a high degree of concurrence between coffee intakes reported by the FFQ and the dietary reference method [31]. Likewise, the correlation between total caffeine calculated by the two methods has been shown to be high [7].

Different aspects of child neurodevelopment such as behaviour, temperament, motor and language skills were accounted for over a large age span from 6 months to 8 years. The outcomes were reported by the mother rather than by health care professionals, which might have influenced the assessment of the child’s abilities and mood. Moreover, the reduction of some of the scales to a few items decreased their reliability, particularly at the 6- and 18-months follow-up assessment. However, the results were fairly robust over the time periods, which strengthens them. The analyses were adjusted for many confounders, which were based on detailed reports, in an attempt to minimize residual confounding.

In this study, we chose to only include full-term pregnancies (> week 37). The rationale for this was the nature of the data; the questionnaires regarding developmental outcomes are administrated strictly according to chronological age and do not take preterm birth into account. This makes interpretation difficult in case of preterm-born children. However, no association between caffeine intake during pregnancy and gestational length or preterm delivery was found in our previous study [7].

## Conclusion

A few associations between caffeine intake and adverse neurodevelopment outcomes were found, but none of them were persistent throughout all ages or different sources or cut-offs of caffeine. Thus, in summary, low to moderate maternal caffeine intake during the first half of the pregnancy was not associated with any consistent, long-term effects on the neurodevelopment in term-born children up to 8 years of age. There were few participants with high caffeine consumption (> 300 mg/day) and we can thus not draw any conclusions regarding the possible effect of high maternal caffeine intake on child neurodevelopment. These results are of clinical importance and based on our findings, there is no need to restrict caffeine consumption during pregnancy further than the current recommendations with regard to the child’s neurodevelopment.

## Electronic supplementary material

Below is the link to the electronic supplementary material.

Supplementary material 1 (DOCX 22 kb)

Supplementary material 2 (DOCX 55 kb)

## Abbreviations

ADHD: Attention deficit hyperactivity disorder
ASQ: The ages and stages questionnaire
CBCL: Child behaviour checklist
CCC-2: The children’s communication checklist-2
CDI: The child development inventory
CI: Confidence interval
EAS: Emotionality, activity and shyness temperament questionnaire
FFQ: Food frequency questionnaire
ICQ: The infant characteristics questionnaire (fussy/difficult subscale)
ITSEA: Infant–Toddler social and emotional assessment
MBRN: Medical birth registry of Norway
MoBa: The Norwegian Mother, Father and Child Cohort Study
OR: Odds ratio
RS-DBD: Parent/teacher rating scales for disruptive behaviour disorders
SCARED: Screen for child anxiety related disorders
SD: Standard deviation
SGA: Small for gestational age
SMFQ: The short moods and feeling questionnaire

## References

[1] Andersson HC, Hallström H, Kihlman BA (2004). Intake of caffeine and other methylxanthines during pregnancy and risk for adverse effects in pregnant women and their foetuses.

[2] Livsmedelsverket. Livsmedel och innehåll; koffein (2015) http://www.livsmedelsverket.se/livsmedel-och-innehall/kosttillskott/amnen-i-kosttillskott/koffein. Accessed 10 Jan 2016

[3] American College of Obstetricians and Gynecologists, ACOG Committee Opinion no. 462 (2010). Moderate caffeine consumption during pregnancy. Obstet Gynecol.

[4] Aldridge A, Aranda JV, Neims AH (1979). Caffeine metabolism in the newborn. Clin Pharmacol Ther.

[5] Aldridge A, Bailey J, Neims AH (1981). The disposition of caffeine during and after pregnancy. Semin Perinatol.

[6] Poole R, Kennedy OJ, Roderick P, Fallowfield JA, Hayes PC, Parkes J (2017). Coffee consumption and health: umbrella review of meta-analyses of multiple health outcomes. BMJ (Clin Res Ed).

[7] Sengpiel V, Elind E, Bacelis J (2013). Maternal caffeine intake during pregnancy is associated with birth weight but not with gestational length: results from a large prospective observational cohort study. BMC Med.

[8] Hoyt AT, Browne M, Richardson S, Romitti P, Druschel C (2014). National Birth Defects Prevention Study. Maternal caffeine consumption and small for gestational age births: results from a population-based case-control study. Matern Child Health J.

[9] Barker DJ (1995). Fetal origins of coronary heart disease. BMJ.

[10] Yi KH, Yi YY, Hwang IT (2016). Behavioral and intelligence outcome in 8- to 16-year-old born small for gestational age. Korean J Pediatr.

[11] Pallotto EK, Kilbride HW (2006). Perinatal outcome and later implications of intrauterine growth restriction. Clin Obstet Gynecol.

[12] Kirkinen P, Jouppila P, Koivula A, Vuori J, Puukka M (1983). The effect of caffeine on placental and fetal blood flow in human pregnancy. Am J Obstet Gynecol.

[13] Hughes RN, Beveridge IJ (1991). Behavioral effects of exposure to caffeine during gestation, lactation or both. Neurotoxicol Teratol.

[14] Nakamoto T, Roy G, Gottschalk SB, Yazdani M, Rossowska M (1991). Lasting effects of early chronic caffeine feeding on rats’ behavior and brain in later life. Physiol Behav.

[15] Souza AC, Souza A, Medeiros LF (2015). Maternal caffeine exposure alters neuromotor development and hippocampus acetylcholinesterase activity in rat offspring. Brain Res.

[16] Bjorklund O, Kahlstrom J, Salmi P, Fredholm BB (2008). Perinatal caffeine, acting on maternal adenosine A (1) receptors, causes long-lasting behavioral changes in mouse offspring. PLoS ONE.

[17] Silva CG, Metin C, Fazeli W (2013). Adenosine receptor antagonists including caffeine alter fetal brain development in mice. Sci Transl Med.

[18] Galera C, Bernard JY, van der Waerden J (2015). Prenatal caffeine exposure and child IQ at age 5.5 years: the EDEN mother-child cohort. Biol Psychiatry.

[19] Klebanoff MA, Keim SA (2015). Maternal caffeine intake during pregnancy and child cognition and behavior at 4 and 7 years of age. Am J Epidemiol.

[20] Bekkhus M, Skjothaug T, Nordhagen R, Borge AI (2010). Intrauterine exposure to caffeine and inattention/overactivity in children. Acta Paediatr.

[21] Barr HM, Streissguth AP (1991). Caffeine use during pregnancy and child outcome: a 7-year prospective study. Neurotoxicol Teratol.

[22] Loomans EM, Hofland L, van der Stelt O (2012). Caffeine intake during pregnancy and risk of problem behavior in 5- to 6-year-old children. Pediatrics.

[23] Linnet KM, Wisborg K, Secher NJ (2009). Coffee consumption during pregnancy and the risk of hyperkinetic disorder and ADHD: a prospective cohort study. Acta Paediatr.

[24] Chiu YN, Gau SS, Tsai WC, Soong WT, Shang CY (2009). Demographic and perinatal factors for behavioral problems among children aged 4-9 in taiwan. Psychiatry Clin Neurosci.

[25] Hvolgaard Mikkelsen S, Obel C, Olsen J, Niclasen J, Bech BH (2017). Maternal caffeine consumption during pregnancy and behavioral disorders in 11-year-old offspring: a danish national birth cohort study. J Pediatr.

[26] Norwegian Institute of Public Health (2015) The Norweigan Mother and Child Cohort Study. http://www.fhi.no/eway/default.aspx?pid=240&trg=MainContent_6894&Main_6664=6894:0:25,7372:1:0:0:::0:0&MainContent_6894=6706:0:25,7375:1:0:0:::0:0. Accessed 17 Jan 2016

[27] Irgrens LM (2000). The Medical Birth Registry of Norway, epidemiological research and surveillance throughout 30 years. Acta Obstet Gynecol Scand.

[28] Magnus P, Birke C, Vejrup K (2016). Cohort profile update: the norwegian mother and child cohort study (MoBa). Int J Epidemiol.

[29] Meltzer HM, Brantsaeter AL, Ydersbond TA, Alexander J, Haugen M (2008). Methodological challenges when monitoring the diet of pregnant women in a large study: experiences from the norwegian mother and child cohort study (MoBa). Matern Child Nutr.

[30] Brantsaeter AL, Haugen M, Alexander J, Meltzer HM (2008). Validity of a new food frequency questionnaire for pregnant women in the norwegian mother and child cohort study (MoBa). Matern Child Nutr.

[31] Brantsaeter AL, Haugen M, Hagve TA (2007). Self-reported dietary supplement use is confirmed by biological markers in the norwegian mother and child cohort study (MoBa). Ann Nutr Metab.

[32] EFSA Panel on Dietetic Products NaA (2015). Scientific opinion on the safety of caffeine. EFSA J.

[33] Bates JE, Freeland CA, Lounsbury ML (1979). Measurement of infant difficultness. Child Dev.

[34] Achenbach TM (1992). Manual for the child behavior checklist/2-3 and 1992 profile.

[35] Carter AS, Briggs-Gowan MJ, Jones SM, Little TD (2003). The infant-toddler social and emotional assessment (ITSEA): factor structure, reliability, and validity. J Abnorm Child Psychol.

[36] Buss AH, Plomin R (1984). Temperament: Early developing personality traits.

[37] Angold A, Costello EJ (ed) (1987) Mood and feelings questionnaire (MFQ). Durham Duke University Development Epidemiology Program

[38] Angold A, Costello EJ, Messer SC, Pickles A, Winder F, Silver D (1995). The development of a short questionnaire for use in epidemiological studies of depression in children and adolescents. Int J Methods Psychiatr Res.

[39] Birmaher B, Khetarpal S, Brent D (1997). The screen for child anxiety related emotional disorders (SCARED): scale construction and psychometric characteristics. J Am Acad Child Adolesc Psychiatry.

[40] Birmaher B, Brent DA, Chiappetta L, Bridge J, Monga S, Baugher M (1999). Psychometric properties of the screen for child anxiety related emotional disorders (SCARED): a replication study. J Am Acad Child Adolesc Psychiatry.

[41] Silva RR, Alpert M, Pouget E (2005). A rating scale for disruptive behavior disorders, based on the DSM-IV item pool. Psychiatr Q.

[42] Squires J, Potter D, Bricker L (1999). The ASQ user’s guide second edition ed. Paul H.

[43] Richter J, Janson H (2007). A validation study of the norwegian version of the ages and stages questionnaires. Acta Paediatr.

[44] Ireton H, Thwing E, Currier SK (1977). Minnesota child development inventory: identification of children with developmental disorders. J Pediatr Psychol.

[45] Bishop DVM (2003). Children’s communication checklist-2.

[46] Bishop DVM (2006). Children’s communication checklist-2 (U.S. edition).

[47] Halsey LG (2019). The reign of the p-value is over: what alternative analyses could we employ to fill the powervacuum?. Biol Lett.

[48] Tollanes MC, Strandberg-Larsen K, Eichelberger KY (2016). Intake of caffeinated soft drinks before and during pregnancy, but not total caffeine intake, is associated with increased cerebral palsy risk in the norwegian mother and child cohort study. J Nutr.

[49] Englund-Ogge L, Brantsaeter AL, Haugen M (2012). Association between intake of artificially sweetened and sugar-sweetened beverages and preterm delivery: a large prospective cohort study. Am J Clin Nutr.

[50] Hernandez-Diaz S, Schisterman EF, Hernan MA (2006). The birth weight “paradox” uncovered?. Am J Epidemiol.

