# Supplementary material for: Maternal caffeine intake during pregnancy and child neurodevelopment up to eight years of age—Results from the Norwegian Mother, Father and Child Cohort Study
Source: Eur J Nutr. 2020 May 26;60(2):791–805. doi: 10.1007/s00394-020-02280-7 (PMC7900051; doi:10.1007/s00394-020-02280-7)
Supplement: Supplementary file 1 — Supplementary material 1 (DOCX 22 kb) [file 394_2020_2280_MOESM1_ESM.docx]

## **Appendix.**

## Questionnaires Behavior and Temperament

The Infant Characteristics Questionnaire (Fussy/Difficult Subscale) evaluates the child’s fussiness at 6 months of age. The mother rates her child’s mood by answering statements on a 7 points scale from “totally disagree” to “totally agree”, for example “Your child whimpers and cries a lot” [1].

The Child Behavior Checklist (CBCL) is used for collecting data regarding behavior and manner divided into internalizing (anxiety/depression) and externalizing (attention/aggression) difficulties. It contains statements that the mother ranks from “not true”, “somewhat or sometimes true” to “very or often true” [2]. The CBCL was part of the MoBa questionnaires at 18 months, 3 and 5 years of age. At 3 years of age, a few items from Infant-Toddler Social and emotional assessment (ITSEA) were added to make the scales more reliable. The ITSEA is used for assessing behavior concerning four main domains: externalizing, internalizing, dysregulation and competencies [3].

The Emotionality, Activity and Shyness Temperament Questionnaire (EAS) captures the child’s temperament; activity level, emotionality (irritability/anger), sociability (positive affect, including approach), and shyness (fear). The mothers grade different statements (for example “Your child prefers playing with others rather than alone”) according to five categories (from “very typical” to “not at all typical”) [4]. The EAS part of the MoBa questionnaires at 18 months, 3 and 5 years of age.

The Short Mood and Feelings Questionnaire is based on DSM-III_R criteria for depression. There is a short form with 13 questions concerning the child’s mood for the last two weeks (for example “didn’t enjoy anything at all” or “felt lonely”) which was part of the MoBa 8-year questionnaire. The questions were answered by the parents with the following options: “not true”, “sometimes true”, “true” [5-6].

Screen for child anxiety disorders (SCARED) is aimed at capturing anxiety disorders including panic disorder, general anxiety, social phobia, separation anxiety and school phobia. A short version with 5 items is used in MoBa and the mothers answer the questions using a 3-point scale. The scale was part of the MoBa 8 years questionnaire [7-8].

Parent/teacher rating scales for Disruptive behaviors (RS-DBD) is based on three aspects; ADHD symptoms, oppositional defiant behavior and items related to conduct disorder. The questions were rated by the parents on a four-point scale (1=never/rarely, 2=sometimes, 3=often and 4=very often). The scale was part of the MoBa 8 years questionnaire [9].

## Questionnaires Motor and Language development

The Ages and Stages Questionnaire (ASQ) holds information regarding gross and fine motor skills and communication/language development. Each question regarding the child’s development (for example “Does your child walk well and seldom fall?”) is answered with “yes”, “sometimes” or “not yet” [10-11]. The ASQ was part of the MoBa 18 months, 3 and 5 years follow-up questionnaires.

The Child Development Inventory (CDI) is used for identification of developmental disorders including fine and gross motor development and was part of the MoBa 5-year follow-up questionnaire [12].

The Children’s Communication Checklist-2 (CCC-2) measures different aspects of communication skills. A short version including 13-items (CCC-S) with addition of 3 items was part of the MoBa 8-year follow-up questionnaire with the aim to screen for speech, language and communication difficulties [13-14].

The numbers below represent the questions chosen for each domain which are found in the Moba questionnaires, including reliability score, available at: [www.fhi.no/en/studies/moba/for-forskere-artikler/questionnaires-from-moba/](http://www.fhi.no/en/studies/moba/for-forskere-artikler/questionnaires-from-moba/)

**6 months** Instrument documentation Q4

*ICQ*

DD386-389, DD391-393

**18 months** Instrument documentation Q5

Motor: *ASQ*

Gross motor EE406-408

Fine motor EE409-411

Language: *ASQ*

EE403-405

Behavior: *CBCL*

Externalizing EE435, EE442, EE446, EE447, EE448, EE903, EE904

Internalizing EE438, EE39, EE49, EE906, EE907, EE908, EE909

Temperament: *EAS*

Negative Emotionality E416, E422, E425

Activity EE417, EE19, EE23

Sociability EE18, EE24

Shyness EE20, EE21, EE26

**3 years** Instrument documentation Q6

Motor: *ASQ*

Gross motor GG222, GG223

Fine motor GG224, GG225

Language: *ASQ*

GG237-242

Behavior: *CBCL*

Externalizing GG314-316, GG326-327, GG329, GG331

Internalizing GG299, GG313, GG317, GG321, GG328, GG336, GG348

GG357, GG359, GG364, GG366

Temperament: *EAS*

Negative Emotionality GG299, GG305, GG308

Activity GG300, GG302, GG306

Sociability GG301, GG307, GG310

Shyness GG303, GG304, GG309

**5 years** Instrument documentation Q-5year

Motor: *CDI*

Gross motor LL265-269

Fine motor LL270-274

Language: *ASQ*

LL175-80

Behavior: *CBCL*

Externalizing LL302-304, LL307, LL308, LL313, LL314, LL316

Internalizing LL301, LL305, LL306, LL309, LL312, LL315, LL317, LL320-322, LL325

Temperament: *EAS*

Negative Emotionality LL279-281

Activity LL276-278

Sociability LL285-287

Shyness LL282-284

**8 years** Instrument documentation Q-8year

Motor: NN37, NN38

Language: *CCC-2*

NN211-226

Behavior: *Disruptive behaviour*

Oppositional defiant NN 137-144 (alpha 0.84)

Conduct NN111-118 (alpha 0.85)

ADHD related symptoms NN 119-136 (alpha 0.90)

Temperament

*SMFQ* NN 68-80 (alpha 0.79)

*Screen for anxiety disorder* NN145-149 (alpha 0.44)

References

1. Bates JE, Freeland CA, Lounsbury ML (1979) Measurement of infant difficultness. Child Dev 50 (3):794-803.

2. Achenbach TM (1992) Manual for the child behavior checklist/2-3 and 1992 profile Burlington, VT: University of Vermont Department of Psychiatry

3. Carter AS, Briggs-Gowan MJ, Jones SM, Little TD (2003) The infant-toddler social and emotional assessment (ITSEA): Factor structure, reliability, and validity. J Abnorm Child Psychol 31(5):495-514.

4. Buss AH, Plomin R (1984) Temperament: Early developing personality traits. Hillsdale, NJ

5. Angold A, & Costello, E.J., ed (1987) Mood and feelings questionnaire (MFQ). Durham Duke University Development Epidemiology Program

6. Angold A., Costello, E.J., Messer, S.C., & Pickles, A., Winder, F., & Silver, D (1995) The development of a short questionnaire for use in epidemiological studies of depression in children and adolescents. International Journal of Methods in Phychiatric Research (5):237-249.

7. Birmaher B, Khetarpal S, Brent D, et al (1997) The screen for child anxiety related emotional disorders (SCARED): Scale construction and psychometric characteristics. J Am Acad Child Adolesc Psychiatry 36(4):545-553.

8. Birmaher B, Brent DA, Chiappetta L, Bridge J, Monga S, Baugher M (1999) Psychometric properties of the screen for child anxiety related emotional disorders (SCARED): A replication study. J Am Acad Child Adolesc Psychiatr*y* 38(10):1230-1236.

9. Silva RR, Alpert M, Pouget E, et al (2005) A rating scale for disruptive behavior disorders, based on the DSM-IV item pool. Psychiatr Q 76(4):327-339.

10. Squires J, Potter D, Bricker L (1999) The ASQ user’s guide Second Edition ed. Paul H. Brookes Publishing Co, Baltimore

11. Richter J, Janson H (2007) A validation study of the norwegian version of the ages and stages questionnaires. Acta Paediatr 96(5):748-752.

12. Ireton,Harold, Thwing,Edward, Currier S,K (1977) Minnesota child development inventory: Identification of children with developmental disorders. Journal of Pediatric Psychology 2(1):18-22.

13. Bishop DVM (2003) Children's communication checklist-2. Pearsson, London

14. Bishop DVM (2006) Children's communication checklist-2 (U.S. edition) NY: The Psychological Corporation, New York
