# Supplementary material for: Maternal caffeine intake during pregnancy and child neurodevelopment up to eight years of age—Results from the Norwegian Mother, Father and Child Cohort Study
Source: Eur J Nutr. 2020 May 26;60(2):791–805. doi: 10.1007/s00394-020-02280-7 (PMC7900051; doi:10.1007/s00394-020-02280-7)
Supplement: Supplementary file 2 — Supplementary material 2 (DOCX 55 kb) [file 394_2020_2280_MOESM2_ESM.docx]

|  | | | | | Total caffeine intake  pre-pregnancy  mg/day | Total caffeine intake  Week 17  mg/day | Total caffeine intake  Week 30  mg/day |
| --- | --- | --- | --- | --- | --- | --- | --- |
| Characteristic | Category | % | | Count | Median  (25^th^; 75^th^ percentile) | Median  (25^th^; 75^th^ percentile) | Median  (25^th^; 75^th^ percentile) |
| Total Cohort |  | |  | 64 189 | 172 (52; 353) | 62 (19;46) | 80 (26;176) |
| Maternal age at delivery (years)* | <25 | | 11.3 | 7263 | 136 (37;313) | 52 (0; 123) | 69 (23; 155) |
|  | 25-29 | | 34.0 | 21833 | 97 (24;371) | 44 (0; 113) | 59 (0; 135) |
|  | 30-34 | | 42.6 | 27357 | 199 (64;371) | 72 (22; 158) | 91 (28;187) |
|  | 35+ | | 12.1 | 7736 | 254 (100;437) | 99 (29; 205) | 107 (31; 215) |
| Maternal education level (years)* | =<12 | | 30.6 | 19638 | 143 (42; 353) | 64 (19; 155) | 82 (25; 186) |
|  | 13-16 | | 41.8 | 26833 | 161 (46; 344) | 57 (15; 136) | 77 (25; 169) |
|  | 17+ | | 25.5 | 16358 | 207 (77; 359) | 67 (22; 147) | 84 (27; 171) |
|  | Missing data | | 2.1 | 1360 | 172 (48; 359) | 61 (0; 154) | 71 (22; 173) |
| Marital status | married/cohabitant | | 96.2 | 61750 | 172 (52; 352) | 62 (19; 145) | 80 (26; 175) |
|  | not married/ cohabitant | | 3.8 | 2439 | 175 (46; 391) | 74 (18; 179) | 81 (19; 193) |
| Alcohol intake  (units/week) * | no | | 89.0 | 57107 | 160 (47; 341) | 58 (14; 136) | 74 (24; 166) |
|  | <0.5 | | 9.2 | 5929 | 245 (105; 420) | 111 (40; 210) | 123 (49; 224) |
|  | >0.5 | | 1.8 | 1153 | 293 (152; 489) | 151 (70; 278) | 158 (61; 293) |
| Smoking habits* | never | | 92.0 | 58709 | 163 (49; 335) | 58 (16; 134) | 75 (25; 163) |
|  | occasionally | | 2.7 | 1731 | 278 (91; 516) | 126 (49; 255) | 141 (51; 282) |
|  | daily | | 5.3 | 3387 | 363 (131; 649) | 195 (81; 365) | 195 (71; 375) |
| Nausea during pregnancy* | no | | 88.7 | 56946 | 176 (53; 356) | 64 (20; 150) | 83 (26; 179) |
|  | yes | | 11.3 | 7243 | 137 (43; 322) | 50 (0; 117) | 66 (23; 151) |
| BMI  prior to pregnancy  kg/m^2^* | <18,5 | | 3.0 | 1886 | 190 (56; 373) | 64 (20; 154) | 84 (26; 171) |
|  | 18,5-24,9 | | 66.2 | 42309 | 185 (63; 398) | 79 (0; 188) | 95 (26; 206) |
|  | 24,9-29,9 | | 21.6 | 13794 | 158 (47; 331) | 58 (19; 135) | 78 (24; 166) |
|  | 30+ | | 9.3 | 5914 | 115 (37; 263) | 50 (16; 110) | 64 (22; 134) |
| Income of >=300,000 NOK/year* | None in the household, | | 27.6 | 17705 | 144 (42; 339) | 58 (15; 141) | 76 (24; 174) |
|  | One in the household | | 41.1 | 26368 | 162 (48; 344) | 61 (17; 143) | 79 (25; 176) |
|  | Both in the household | | 28.6 | 18326 | 208 (71; 374) | 71 (23; 153) | 88 (27; 176) |
|  | Missing data | | 2.8 | 1790 | 143 (39; 344) | 63 (0; 150) | 68 (0; 171) |
| Tertiles of fiber intake* | 1 | | 33.3 | 21394 | 138 (42; 312) | 58 (17; 134) | 73 (24; 161) |
|  | 2 | | 33.3 | 21388 | 176 (54; 351) | 62 (19; 145) | 81 (26; 174) |
|  | 3 | | 33.3 | 21397 | 202 (61; 389) | 68 (20; 160) | 88 (27; 192) |
| Sex of baby | Boy | | 50.7 | 32572 | 171 (52; 348) | 63 (19; 146) | 80 (26; 175) |
|  | Girl | | 49.3 | 31617 | 172 (51; 357) | 62 (19; 147) | 80 (25; 176) |

**Supplementary table1**. Total caffeine reported at pre-pregnancy, week 17 and week 30 during pregnancy, according to maternal characteristics, n=64 189 from the Norwegian Mother and Child cohort.

BMI: Body mass index. NOK: Norwegian kroner

**Supplementary table 2.** Adjusted Odds Ratios for child temperament and behavior outcomes according to caffeine intake from different sources at different ages from 18 months to 8 years of age.

| Caffeine intake (100 mg/day) | Negative  emotionality | | High  Activity | | Shyness | | Low  Sociability | | Internalizing  behaviour | | | | | Externalizing behaviour | | | | |
| --- | --- | --- | --- | --- | --- | --- | --- | --- | --- | --- | --- | --- | --- | --- | --- | --- | --- | --- |
| 18 months | OR | 95% CI | OR | 95% CI | OR | 95% CI | OR | 95% CI | OR | | 95% CI | | | OR | | | 95% CI | |
| Coffee | 1.014 | 0.971-1.060 | **1.039** | 1.004-1.076 | 0.981 | 0.944-1.021 | 1.001 | 0.962-1.043 | 0.974 | | 0.931-1.018 | | | 0.978 | | | 0.939-1.019 | |
| Tea | 1.016 | 0.902-1.145 | 0.969 | 0.877-1.070 | 1.097 | 0.993-1.211 | **1.171** | 1.056-1.298 | **1.190** | | 1.065-1.329 | | | 1.098 | | | 0.986-1.224 | |
| Soft drinks | 1.107 | 0.980-1.251 | **1.240** | 1.125-1.368 | 0.927 | 0.824-1.044 | 0.966 | 0.854-1.091 | 0.945 | | 0.828-1.079 | | | 1.007 | | | 0.899-1.128 | |
| Chocolate | **2.745** | 1.565-4.816 | 0.942 | 0.565-1.571 | **1.910** | 1.145-3.186 | **0.317** | 0.172-0.585 | **2.338** | | 1.325-4.123 | | | 1.254 | | | 0.729-2.158 | |
|  |  | | | | | | | | | | | | | | | | | |
| 3 yrs | OR | 95% CI | OR | 95% CI | OR | 95% CI | OR | 95% CI | OR | | 95% CI | | | OR | | | 95% CI | |
| Coffee | **1.049** | 1.004-1.097 | 1.039 | 0.978-1.103 | 0.980 | 0.934-1.029 | 0.973 | 0.983-1.029 | 0.955 | | 0.909-1.003 | | | 1.014 | | | 0.966-1.064 | |
| Tea | 0.984 | 0.871-1.112 | 1.056 | 0.894-1.246 | 1.001 | 0.885-1.131 | 1.122 | 0.977-1.288 | 1.026 | | 0.906-1.162 | | | 1.099 | | | 0.966-1.250 | |
| Soft drinks | 1.118 | 0.985-1.268 | **1.443** | 1.245-1.671 | 0.947 | 0.822-1.091 | 1.075 | 0.923-1.252 | 1.046 | | 0.917-1.193 | | | 1.103 | | | 0.968-1.257 | |
| Chocolate | 1.467 | 0.804-2.675 | 2.192 | 0.962-4.993 | 1.469 | 0.786-2.746 | 0.931 | 0.434-1.997 | **1.873** | | 1.019-3.441 | | | 1.032 | | | 0.535-1.990 | |
|  |  | | | | | | | | | | | | | | | | |  |
| 5 yrs | OR | 95% CI | OR | 95% CI | OR | 95% CI | OR | 95% CI | OR | 95% CI | | | OR | | | 95% CI | |  |
| Coffee | 0.992 | 0.935-1.052 | **1.076** | 1.022-1.133 | 0.890 | 0.825-0.961 | 0.970 | 0.906-1.040 | 0.952 | 0.895-1.013 | | | 1.009 | | | 0.954-1.069 | |  |
| Tea | 1.040 | 0.892-1.213 | 1.025 | 0.884-1.188 | 0.796 | 0.653-0.971 | 0.976 | 0.811-1.176 | 1.059 | 0.904-1.242 | | | 1.053 | | | 0.901-1.230 | |  |
| Soft drinks | 0.868 | 0.721-1.044 | **1.335** | 1.155-1.543 | 1.033 | 0.854- 1.236 | 0.990 | 0.808-1.214 | 0.920 | 0.767-1.103 | | | 0.918 | | | 0.771-1.092 | |  |
| Chocolate | 0.954 | 0.441-2.062 | 1.475 | 0.702- 3.015 | 1.191 | 0.476-2.981 | 0.540 | 0.205-1.423 | 1.071 | 0.487-2.355 | | | 1.116 | | | 0.518-2.406 | |  |
|  | | | | | | | | | | | | | | | | | |  |
|  | Conduct  Disorder | | ADHD-related symptoms | | Oppositional  defiant | | Depression  symptoms | | Scared/Anxiety problems | | | | | |  | | |  |
| 8 yrs | OR | 95% CI | OR | 95% CI | OR | 95% CI | OR | 95% CI | OR | | | 95% CI | | |  | | |  |
| Coffee | 1.040 | 0.963-1.122 | 1.023 | 0.951-1.099 | 1.046 | 0.978-1.118 | **1.080** | 1.018-1.145 | 0.976 | | | 0.911-1.046 | | |  |  |  |  |
| Tea | 0.989 | 0.793-1.234 | 0.958 | 0.778-1.180 | 0.827 | 0.677-1.011 | **0.803** | 0.673-0.959 | 0.887 | | | 0.738-1.067 | | |  |  |  |  |
| Soft drinks | 1.003 | 0.786-1.281 | 1.099 | 0.891-1.356 | 0.931 | 0.752-1.153 | 1.018 | 0.848-1.223 | 1.020 | | | 0.833-1.250 | | |  |  |  |  |
| Chocolate | 0.329 | 0.089-1.211 | 1.751 | 0.617-4.970 | 1.001 | 0.363-2.757 | 0.606 | 0.244-1.504 | 2.235 | | | 0.865-5.776 | | |  |  |  |  |

Odds ratios for caffeine from different sources and child behavior and temperament outcomes at different ages.

Adjusted for: maternal age, smoking, alcohol intake, marital status, baby’s gender, household income, maternal education, dietary fiber, total energy intake, nausea, maternal mental health and mutually adjusted for the other caffeine sources.

OR=odds ratio. CI=confidence interval. OR (CI) from logistic regressions. The outcomes are based on the CBCL and EAS scales.

**Supplementary table 3.** Adjusted Odds Ratios for child language and motor difficulties according to caffeine intake from different sources at different ages between 18 months and 8 years of age.

| Caffeine intake (100 mg/day) | Gross motor | | Fine motor | | Language | |
| --- | --- | --- | --- | --- | --- | --- |
| 18 months | OR | 95% CI | OR | 95% CI | OR | 95% CI |
| Coffee | 1.036 | 0.994-1.080 | 0.987 | 0.953-1.023 | 1.022 | 0.984-1.061 |
| Tea | **1.192** | 1.070-1.328 | 1.032 | 0.938-1.134 | **1.241** | 1.126-1.367 |
| Soft drinks | 1.055 | 0.931-1.195 | 0.974 | 0.876-1.082 | 1.009 | 0.901-1.130 |
| Chocolate | 1.307 | 0.725-2.356 | 0.899 | 0.547-1.477 | 1.310 | 0.769-2.230 |
| 3 yrs | OR | 95% CI | OR | 95% CI | OR | 95% CI |
| Coffee | 0.959 | 0.886-1.038 | 0.965 | 0.922-1.009 | 0.998 | 0.944-1.056 |
| Tea | 0.996 | 0.820-1.211 | 0.975 | 0.870-1.094 | 1.135 | 0.979-1.316 |
| Soft drinks | 0.761 | 0.586-0.988 | 0.939 | 0.820-1.075 | **1.212** | 1.047-1.402 |
| Chocolate | 0.441 | 0.149-1.306 | 0.815 | 0.443-1.499 | 0.296 | 0.126-0.691 |
| 5 yrs | OR | 95% CI | OR | 95% CI | OR | 95% CI |
| Coffee | 0.990 | 0.917-1.070 | 0.954 | 0.892-1.020 | 0.911 | 0.849-0.978 |
| Tea | 0.993 | 0.813-1.211 | **1.226** | 1.038-1.448 | 1.063 | 0.889-1.271 |
| Soft drinks | 0.824 | 0.639-1.064 | 1.118 | 0.929-1.346 | **1.299** | 1.095-1.540 |
| Chocolate | 0.828 | 0.293-2.339 | 1.269 | 0.523-3.079 | 1.172 | 0.478-2.869 |
|  | Motor | |  | | Language | |
| 8 yrs | OR | 95% CI |  |  | OR | 95% CI |
| Coffee | 0.939 | 0.792-1.115 |  |  | 0.982 | 0.919-1.050 |
| Tea | 1.058 | 0.671-1.668 |  |  | 1.079 | 0.902-1.292 |
| Soft drinks | 1.197 | 0.806-1.779 |  |  | 0.919 | 0.745-1.133 |
| Chocolate | 0.610 | 0.056-6.697 |  |  | 0.948 | 0.332-2.707 |

Odds ratios for caffeine from different sources and language and motor difficulties during childhood at different ages. Adjusted for: maternal age, smoking, alcohol intake, marital status, baby’s gender, household income, maternal education, dietary fiber, total energy intake, nausea, maternal mental health and mutually adjusted for the other caffeine sources. OR=odds ratio. CI=confidence interval. OR (CI) from logistic regression. The outcomes are based on the ASQ, CDI and CCC-2 scales.

**Supplementary table 4.** Adjusted Odds Ratios for caffeine intake from different sources and category of caffeine intake and child temperamental outcome at 6 months of age

|  | ICQ/Fuzziness | |
| --- | --- | --- |
| Caffeine intake (mg/day) | OR | 95% CI |
| Total caffeine intake | 1.033 | 0.998-1.069 |
| Coffee* | 1.003 | 0.965-1.043 |
| Tea* | **1.111** | 1.005-1.229 |
| Soft drink* | **1.158** | 1.043-1.285 |
| Chocolate* | **2.049** | 1.464-3.963 |
| Caffeine intake 0-22  (n= 13428) | Ref |  |
| Caffeine intake >22-56 (n=13900) | 1.044 | 0.958-1.137 |
| Caffeine intake >56-200 (n=21136) | 1.066 | 0.983-1.155 |
| Caffeine intake >200-300 (n=3950) | 1.096 | 0.962-1.249 |
| Caffeine intake >300 (n=1714) | 1.186 | 0.989-1.421 |

Odds ratio for maternal caffeine intake from different sources and categories of caffeine intake and temperamental problem at 6 months of age based on Infant Characteristics Questionnaire (ICQ).

Adjusted for: maternal age, smoking, alcohol intake, marital status, baby’s gender, household income, maternal education, dietary fiber, total energy intake, nausea, maternal mental health. *Mutually adjusted for the other caffeine sources. Categories in mg/day. OR=odds ratio. CI=confidence interval. OR (CI) from logistic regression.

**Supplementary table 5.** Adjusted Odds Ratios for child temperament and behavior outcomes according to caffeine intake at different time points during pregnancy

| Caffeine intake  (per 100 mg/day) | Negative  emotionality | | High  Activity | | Shyness | | Low  Sociability | | Internalizing  behaviour | | Externalizing behaviour | | |
| --- | --- | --- | --- | --- | --- | --- | --- | --- | --- | --- | --- | --- | --- |
| 18 months | OR | 95% CI | OR | 95% CI | OR | 95% CI | OR | 95% CI | OR | 95% CI | OR | | 95% CI |
| Caffeine intake pre-pregnancy | 0.997 | 0.981-1.014 | 1.012 | 0.999-1.025 | 0.992 | 0.976-1.007 | 0.997 | 0.982-1.013 | 0.998 | 0.981-1.014 | **0.980** | | 0.964-0.996 |
| Caffeine intake w 17 | 1.001 | 0.978-1.025 | 1.002 | 0.984-1.020 | 1.001 | 0.978-1.024 | 1.007 | 0.988-1.026 | 1.004 | 0.983-1.025 | 0.996 | | 0.971-1.022 |
| Caffeine intake w 30 | 1.015 | 0.997-1.034 | 1.012 | 0.996-1.029 | 0.997 | 0.975-1.019 | 1.005 | 0.985-1.025 | 0.987 | 0.961-1.014 | **1.040** | | 1.020-1.060 |
|  |  | | | | | | | | | | | | |
| 3 yrs | OR | 95% CI | OR | 95% CI | OR | 95% CI | OR | 95% CI | OR | 95% CI | | OR | 95% CI |
| Caffeine intake pre-pregnancy | 1.003 | 0.985-1.021 | 0.998 | 0.973-1.023 | 1.004 | 0.985-1-022 | 0.999 | 0.978-1.020 | 0.996 | 0.978-1.015 | | 1.002 | 0.984-1.020 |
| Caffeine intake w 17 | 0.993 | 0.962-1.026 | 1.035 | 0.999-1.073 | 1.009 | 0.989-1.030 | 1.001 | 0.974-1.030 | 0.997 | 0.969-1.025 | | 1.006 | 0.986-1.026 |
| Caffeine intake w 30 | 1.018 | 0.997-1.039 | 1.010 | 0.980-1.040 | 0.977 | 0.946-1.008 | 1.004 | 0.975-1.033 | 0.998 | 0.971-1.026 | | 1.010 | 0.987-1.034 |
| 5 yrs | OR | 95% CI | OR | 95% CI | OR | 95% CI | OR | 95% CI | OR | 95% CI | | OR | 95% CI |
| Caffeine intake pre-pregnancy | 1.001 | 0.979-1.023 | 1.004 | 0.982-1.027 | 1.012 | 0.984-1.040 | 0.991 | 0.966-1.017 | 1.018 | 0.994-1.043 | | 1.004 | 0.984-1.025 |
| Caffeine intake w 17 | 1.009 | 0.989-1.030 | **1.063** | 1.020-1.109 | **0.904** | 0.849-0.963 | 1.003 | 0.978-1.029 | 0.984 | 0.940-1.031 | | 1.004 | 0.983-1.025 |
| Caffeine intake w 30 | 0.972 | 0.936-1.010 | 0.984 | 0.953-1.016 | 1.004 | 0.969-1.040 | 1.000 | 0.968-1.032 | 0.963 | 0.924-1.004 | | 0.998 | 0.972-1.024 |
|  | | | | | | | | | | | | | |
|  | Conduct Disorder | | ADHD-symptoms | | Oppositional defiant | | Depression symptoms | | Scared/Anxiety | | |  | |
| 8 yrs | OR | 95% CI | OR | 95% CI | OR | 95% CI | OR | 95% CI | OR | 95% CI | |  | |
| Caffeine intake pre-pregnancy | 0.995 | 0.964-1.026 | 1.024 | 0.997-1.053 | 1.015 | 0.987-1.045 | **1.026** | 1.002-1.052 | 1.021 | 0.994-1.050 | |  | |
| Caffeine intake w 17 | 1.000 | 0.968-1.034 | 0.998 | 0.969-1.028 | 0.981 | 0.929-1.036 | 0.987 | 0.949-1.028 | 0.983 | 0.932-1.037 | |  |  |
| Caffeine intake w 30 | 1.044 | 0.999-1.091 | 1.039 | 0.997-1.082 | 1.036 | 0.995-1.079 | **1.040** | 1.003-1.079 | 1.001 | 0.956-1.048 | |  |  |

Odds ratios for caffeine intake at different time points during pregnancy and child behavior and temperament outcomes at different ages. Adjusted for: maternal age, smoking, alcohol intake, marital status, baby’s gender, household income, maternal education, dietary fiber, total energy intake, nausea, maternal mental health and mutually adjusted for each other.

OR=odds ratio. CI=confidence interval. OR (CI) from logistic regressions. The outcomes are based on the CBCL and EAS scales.

**Supplementary table 6**. Adjusted Odds Ratios for child language and motor difficulties according to caffeine intake at different time points during pregnancy

| Caffeine intake  (per 100 mg/day) | Gross motor | | Fine motor | | Language | |
| --- | --- | --- | --- | --- | --- | --- |
| 18 months | OR | 95% CI | OR | 95% CI | OR | 95% CI |
| Caffeine intake pre-pregnancy | 1.000 | 0.983-1.016 | 0.998 | 0.984-1.012 | 1.003 | 0.989-1.018 |
| Caffeine intake w 17 | **1.024** | 1.003-1.047 | **1.021** | 1.001-1.040 | 1.005 | 0.987-1023 |
| Caffeine intake w 30 | 1.008 | 0.988-1.028 | 0.991 | 0.970-1.012 | 1.014 | 0.997-1.031 |
| 3 yrs | OR | 95% CI | OR | 95% CI | OR | 95% CI |
| Caffeine intake pre-pregnancy | 0.980 | 0.948-1.014 | 0.985 | 0.966-1.004 | 1.003 | 0.983-1.024 |
| Caffeine intake w 17 | 0.985 | 0.921-1.054 | 0.977 | 0.941-1.015 | 1.004 | 0.982-1.027 |
| Caffeine intake w 30 | 0.981 | 0.927-1.038 | 1.013 | 0.989-1.037 | 1.020 | 0.996-1.044 |
| 5 yrs | OR | 95% CI | OR | 95% CI | OR | 95% CI |
| Caffeine intake pre-pregnancy | 1.020 | 0.990-1.051 | 0.997 | 0.973-1.021 | 0.976 | 0.952-1.000 |
| Caffeine intake w 17 | 0.980 | 0.923-1.041 | 0.995 | 0.965-1.027 | 1.004 | 0.980-1.028 |
| Caffeine intake w 30 | 0.984 | 0.937-1.033 | 1.013 | 0.988-1.038 | 1.006 | 0.979-1.034 |
|  | Motor | |  | | Language | |
| 8 yrs | OR | 95% CI |  |  | OR | 95% CI |
| Caffeine intake pre-pregnancy | 1.013 | 0.951-1.080 |  |  | **1.025** | 1.002-1.049 |
| Caffeine intake w 17 | 0.961 | 0.845-1.094 |  |  | 0.958 | 0.906-1.012 |
| Caffeine intake w 30 | 1.015 | 0.961-1.072 |  |  | 0.968 | 0.925-1.013 |

Odds ratios for caffeine intake at different time points during pregnancy and language and motor difficulties during childhood at different ages. Adjusted for: maternal age, smoking, alcohol intake, marital status, baby’s gender, household income, maternal education, dietary fiber, total energy intake, nausea, maternal mental health and mutually adjusted for each other. OR=odds ratio. CI=confidence interval. OR (CI) from logistic regressions. The outcomes are based on the ASQ, CDI and CCC-2 scales.

**Supplementary table 7**. Crude Odds Ratios for prenatal intake of soft drinks with and without caffeine and child activity level

|  | Soft drinks  Caffeinated  mg/day | | Soft drinks  Decaffeinated  mg/day | |
| --- | --- | --- | --- | --- |
|  | OR | 95% CI | OR | 95% CI |
| Activity 18 months | **1.011** | 1.008-1.015 | **1.009** | 1.003-1.016 |
| Activity 3 yrs | **1.018** | 1.012-1.023 | **1.017** | 1.007-1.027 |
| Activity 5 yrs | **1.016** | 1.010-1.021 | **1.013** | 1.003-1.023 |

Unadjusted analyses. OR=odds ratio. CI=confidence interval. OR (CI) from logistic regression. The outcomes are based on EAS scales.
